# Highly Sensitive Flexible SERS-Based Sensing Platform for Detection of COVID-19

**DOI:** 10.3390/bios12070466

**Published:** 2022-06-28

**Authors:** Seyyed Mojtaba Mousavi, Seyyed Alireza Hashemi, Vahid Rahmanian, Masoomeh Yari Kalashgrani, Ahmad Gholami, Navid Omidifar, Wei-Hung Chiang

**Affiliations:** 1 Department of Chemical Engineering, National Taiwan University of Science and Technology, Taipei City 106335, Taiwan; kempo.smm@gmail.com; 2 Nanomaterials and Polymer Nanocomposites Laboratory, School of Engineering, University of British Columbia, Kelowna, BC V1V 1V7, Canada; s.a.hashemi0@gmail.com; 3 Centre of Molecular and Macromolecular Studies, Polish Academy of Sciences, Sienkiewicza 112, 90-363 Lodz, Poland; vahid.ra@cbmm.lodz.pl; 4 Biotechnology Research Center, Shiraz University of Medical Science, Shiraz 71468-64685, Iran; masoomeh.yari.72@gmail.com; 5 Department of Pathology, School of Medicine, Shiraz University of Medical Sciences, Shiraz 71468-64685, Iran; omidifarn@sums.ac.ir

**Keywords:** flexible SERS substrates, coronavirus, ultrasensitive detection, biomarker

## Abstract

COVID-19 continues to spread and has been declared a global emergency. Individuals with current or past infection should be identified as soon as possible to prevent the spread of disease. Surface-enhanced Raman spectroscopy (SERS) is an analytical technique that has the potential to be used to detect viruses at the site of therapy. In this context, SERS is an exciting technique because it provides a fingerprint for any material. It has been used with many COVID-19 virus subtypes, including Deltacron and Omicron, a novel coronavirus. Moreover, flexible SERS substrates, due to their unique advantages of sensitivity and flexibility, have recently attracted growing research interest in real-world applications such as medicine. Reviewing the latest flexible SERS-substrate developments is crucial for the further development of quality detection platforms. This article discusses the ultra-responsive detection methods used by flexible SERS substrate. Multiplex assays that combine ultra-responsive detection methods with their unique biomarkers and/or biomarkers for secondary diseases triggered by the development of infection are critical, according to this study. In addition, we discuss how flexible SERS-substrate-based ultrasensitive detection methods could transform disease diagnosis, control, and surveillance in the future. This study is believed to help researchers design and manufacture flexible SERS substrates with higher performance and lower cost, and ultimately better understand practical applications.

## 1. Introduction

Infectious diseases such as coronaviruses (CoV), especially coronavirus disease 2019 (COVID-19), infect and kill millions of people worldwide [1,2]. These viruses are transmitted by individual contact with a contaminated surface and subsequent contact with the mouth or nose, as well as by inhalation of tiny droplets exhaled by infected individuals when they cough or sneeze [3]. Individuals who have influenza present with clinical features of symptomatic COVID-19 infection, including dyspnea, cough, and fever, which can lead to medical complications such as kidney damage and pneumonia. Therefore, a complete diagnosis with sensitive and accurate analytical methods or rapid diagnosis is required for optimal patient therapy [4,5,6]. Significant advances in surface-enhanced Raman spectroscopy (SERS) as a suitable point-of-care testing (POCT), since its introduction in 1973, have been demonstrated for a number of analytes, including viruses [7,8]. The complete molecular composition of microorganisms, bacteria, and viruses is reflected in the Raman spectrum, which includes many different vibrational states of reliable classifiers (proteins, carbohydrates, DNA/RNA, and lipids) as well as nonspecific constituents of species (such as carotenoids). Thus, Raman spectra contain both genetic and phenotypic signatures of the studied microorganisms, bacteria, and viruses, since all cellular components are based on the expression of different parts of the genome [9,10]. One of the features of surface-enhanced Raman spectroscopy (SERS) is the identification of characteristic peaks of the outer-membrane proteins of molecules in the sensitive and rapid detection of various molecules, including viruses such as coronavirus [11]. Other features of SERS for coronavirus detection include the use of electromagnetic-field enhancement, by exciting local-surface plasmon resonances in nanostructured-metal surfaces such as gold or silver [12]. The detection of analytes at very low concentrations, and the performance of assays without pretreatment are among the capabilities of SERS. When used in conjunction with an immunoassay, the technology provides an extremely high level of specificity [13,14]. In addition, multiplex SERS immunoassays can detect a variety of analytes, which significantly increases the versatility of the technique compared to the polymerase chain reaction (PCR), which can only analyze materials containing genetic material [15,16]. Therefore, the most promising option for faster detection of COVID-19 than PCR is the use of surface-enhanced Raman spectroscopy (SERS). The PCR protocol amplifies single-stranded DNA to 100 billion copies after 40 cycles of doubling, to achieve a sufficient fluorescent-signal strength for virus detection, which takes hours. Identification of unique gene sequences and single nucleotide polymorphisms by SERS-amplified signals provides more accurate and cost-effective diagnosis, while DNA amplification from COVID-19 does not require a long time [17].

Among the advantages of SERS over other COVID-19-detection methods is the identification of gene sequences through the commercial availability of multiple Raman dyes, leading to the development of nonoverlapping gene-detection probes. SERS tags in ultrasensitive detection also include antibodies, aptamers, and DNA; the detection of proteins, DNA, and other components is based on SERS tags [18,19]. Other advantages of SERS include the speed of analysis, emergence of robust and commercially available Raman spectrometers, simplicity of sample manipulation, and on-site detection of analytes [20]. Limitations of the SERS method include the need for close contact between the analyte and the amplifier surface, degradation of substrates over time that reduces the signal, limited reuse of substrates, problems with homogeneity and reproducibility of the SERS signal in a substrate, and limited substrate selection for a given analyte [21]. In recent years, the SERS-based ultrasensitive sensing platform has emerged as the most promising option for the detection of selective analytes at the nanostructured level, due to its specificity, high sensitivity, nondestructive sensing, and narrow linewidth. The presence of a target analyte near a metal surface leads to SERS events. Adsorbed targets, incident light, and metal nanoparticles are three important factors in optimizing the SERS measurement model for Raman signals from adsorbed targets. resulting in several great reviews and monographs [22]. Moreover, the composition, size, and morphology of the plasmonic metal nanostructures allow for the configuration of a wide range of optimization parameters. Currently, the design of the SERS platform is guided by the visual application of some commonly accepted rules. Gold and silver metals are widely used to make nanoparticles because the surface transitions cause localized surface-plasmon resonance in the visible region of the electromagnetic spectrum. Gold is used because of its chemical stability, oxidation resistance, and ability to be functionalized with more organic materials. Plasmon resonance of silver nanoparticles occurs at lower wavelengths and with greater intensity than gold nanoparticles. Silver nanoparticles have stronger peaks than gold and are, therefore, more sensitive to the refractive index of the environment [2,23]. This means that silver is the most advanced of the plasmonic materials, although gold is more versatile in practice due to the greater variety of available nanoparticle shapes and higher chemical stability. A simple and effective parameter to optimize SERS is the particle density in SERS substrates [24,25].

The aim of this review study was to review recent advances in the fabrication of highly sensitive flexible-SERS substrates for COVID-19-detection work in this field. That is in addition to techniques for resonance amplification and SERS spectroscopy. Numerous researchers have thoroughly investigated the processes behind the benefits of SERS, high-quality nanostructures for SERS-based detection, the principles of SERS and amplification mechanisms, analyte detection, multiplex analysis for coronavirus detection, and SERS COVID-19 detection, all of which were evaluated.

## 2. Techniques for Resonance Enhancement

When the excitation laser resonates with the electronic transmission of the analyte, the surface-enhanced resonance Raman-scattering (SERRS) effect occurs, as noted by Yuan et al. [26]. Molecular-resonance excitation can increase the effective cross-sectional area of the target analytes by many orders of magnitude. Numerous articles do not distinguish between SERS and SERRS, although both have different properties. Compared with SERS, the SERRS mechanism exhibits a significant improvement. Moreover, the intensity of SERRS is wavelength-dependent. SERRS spectroscopy has evolved to the point where it can identify single resonant molecules, as both the molecular-resonance effect and the surface-plasmon-resonance (SPR) enhancement contribute to the generation of exceptionally strong Raman-scattering signals [27]. Excellent candidates for SERRS research are fluorescent protein molecules (such as proteins, rhodamine 6G (R6G), malachite green, antibodies, purple crystals, etc.) or luminescent dyes due to large Raman-scattering cross-sections and intense optical absorption in the visible region. The single molecule can be uniquely identified by SERRS spectroscopy through initial observations in the visible window. Matching the broad adsorption range of the surface-plasmon resonance (SPR) of aggregated silver colloids, with stimulation by Raman-scattering resonance, is due to the most commonly used substrate, as shown in Table 1. Non-invasive photothermal imaging has gained considerable attention in recent years, due to the cross-sectional area of massive absorption of nanomaterials in the near-infrared region. Despite the minimal energy loss during penetration, NIR light using heat-generating nanomaterials is a good choice for targeting cells, because it does not damage adjacent normal tissue. For example, the fabrication of unique gold nanorods with SERS coding (SPR band, 790 nm) shows high optical absorption at 810 nm for photothermal heating, although it does not fully match the NIR-excitation source at 785 nm [28].

### 2.1. SERS Spectroscopy

**Figure 1:**
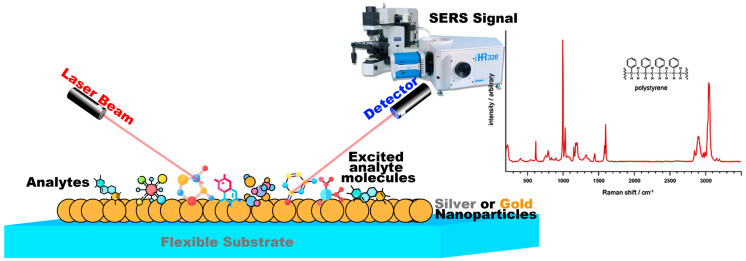
The Raman signal can be amplified further when the roughened metal surface is used in combination with laser light that is matched to the absorption maxima of the molecule. This effect is known as surface-enhanced resonance Raman scattering (SERS), which is shown in the figure.

Surface-enhanced Raman spectroscopy is an extended form of Raman spectroscopy, in which metal nanostructures are used to enhance Raman scattering. In this case, the efficiency of Raman scattering for molecules adsorbed on metal nanostructures increases dramatically, from 10^12^ to 10^14^. A small number of scattered photons are accessible for detection due to the intrinsic weakness of Raman signals, particularly when excited by visible light. Utilizing surface-enhanced Raman scattering is one way to increase weak Raman signals (SERS). SERS employs nanoscale, roughened metal surfaces consisting of gold (Au) or silver (Ag), The schematic representation of SERS can be found in Figure 1. Therefore, surface-enhanced Raman spectroscopy (SERS) is currently a widely used optical tool for the analysis of the molecular components of chemicals and biological samples, with the potential to detect single molecules [34]. The overlap of the excitation wavelength of the Raman laser with the resonance wavelength of the plasmon structure has made it possible to use SERS spectroscopy in sensors and various laboratory analyses. This is because the resonance of the substituted surface plasmons plays an important role in electromagnetic amplification [35,36]. Therefore, the preparation of colloids and metal substrates with the ability to generate and maintain plasmonic effects is of great interest [37,38]. Therefore, the results of SERS spectroscopy depend on the performance of the substrate in signal amplification and its reproducibility [39]. Among the various methods to fabricate a suitable substrate, electrostatic self-assembly of plasmonic nanoparticles, especially gold and silver, on a functionalized substrate is an easy way to obtain a uniform, reproducible, and cost-effective substrate. Different plasmonic nanoparticles can be used depending on laboratory conditions. However, reports show that silver nanoparticles provide the highest Raman-signal enhancement in the visible-wavelength range [16,40]. Considering the influence of nanoparticle size on Raman-signal amplification [41], it is important to choose an appropriate method to prepare nanoparticles of the desired size. Therefore, to prepare silver nanoparticles, a better chemical method was used to obtain nanoparticles of desired size for the SERS substrate. Various probes are used for SERS, among which pigment molecules are the best and most commonly used [42]. The reason for this is the structure of the pigments, which allows them to bind to the surface of the metal nanoparticles. All pigments are water-soluble, so they are suitable for use in the colloids of nanoparticles synthesized in water, as a better chemical process. Moreover, these structures can be used in solid substrates such as self-assembling substrates. On the other hand, the pigments produce very intense background fluorescence at the visible wavelength of the laser, which interferes with the Raman signals. Numerous factors, such as the type of pigment, the distance between the nanoparticles and the pigment molecules, and the dimensions of the substrate nanoparticles used, affect the Raman spectrum obtained [43,44]. By using a suitable substrate, the Raman signal can be enhanced, and the fluorescence effects of the pigments can be eliminated [45].

### 2.2. High-Quality Nanostructures for SERS-Based Detection

The SPR-enhancement property, which is very important for ultrasensitive diagnosis, depends strongly on the type of nanoparticles used. Variations in the properties of the nanostructures, including composition, stability, shape, dielectric parameters, size, and surface modification, contribute significantly to the generation of SPR-induced EM enhancement. In recent decades, various types of prominent nanostructures have been designed and developed for SERS-based ultrasensitive diagnostic studies, including aggregated nanostructures, self-assembled nanostructures, uniform-plasmonic nanostructures, alloyed nanostructures, magnetic nanostructures, carbon-based materials, three-dimensional X-ray structures, silicon-based nanomaterials, and metal–organic frameworks [46]. Nanostructured materials often exhibit new physical and chemical properties relative to the bulk state. Due to the properties of nanomaterials and their special applications, various types of metal nanostructures, including nanoparticles, single nanoparticles, composite nanoparticles, and nanoparticle clusters, are used in SERS. Usually metal nanoparticles of gold, silver, and copper are used as active ingredients in SERS to detect COVID-19, due to their plasmonic peak in the visible region. These metal nanoparticles provide localized surface-plasmon resonance, which creates a large localized electromagnetic field for the detection of COVID-19. Metal nanoparticles with different sizes and shapes create distinct LSPR properties and dramatically increase the effect of SERS [47].

To get a better idea of the reinforcement properties, the SERS-reinforcement properties of the different nanostructures are discussed in more detail in the following sections. Aggregated nanostructures serve as initial sensing platforms for ultrasensitive detection based on SERS. Usually, most substrates used for simple silver aggregates are prepared by boiling silver nitrate with sodium-citric acid (Lee-Meisel technique) [48]. Ideally, the broad absorption range of nanoaggregates under laser irradiation is in the size range of 10–150 nm, to obtain and enhance significant metal SPR for single-molecule detection. The SPR-adsorption band of silver nanoparticles has a significant impact on the generation of SERS effects, when used to efficiently activate metallic SPR. Activation of silver colloids by the analytes themselves occurs through the modulation of colloidal aggregation states, when the target analytes contain active atoms such as nitrogen or sulphur. Salt-induced aggregation (sodium chloride or potassium chloride) is able to solve the problem, when the target molecules are unable to produce colloidal aggregation due to the altered surface charge [49].

SERS-based detection of single molecules showed that the sensitivity of detection by addition of activation solutions strongly depends on factors such as random adsorption sites (hot spots) of the nanoaggregates. Recently, it was reported that an ultrasensitive method for the detection of ozone was developed using accumulated gold nanoparticles as SERS substrate [50,51].

### 2.3. Principles of SERS and Enhancement Mechanisms

**Figure 2:**
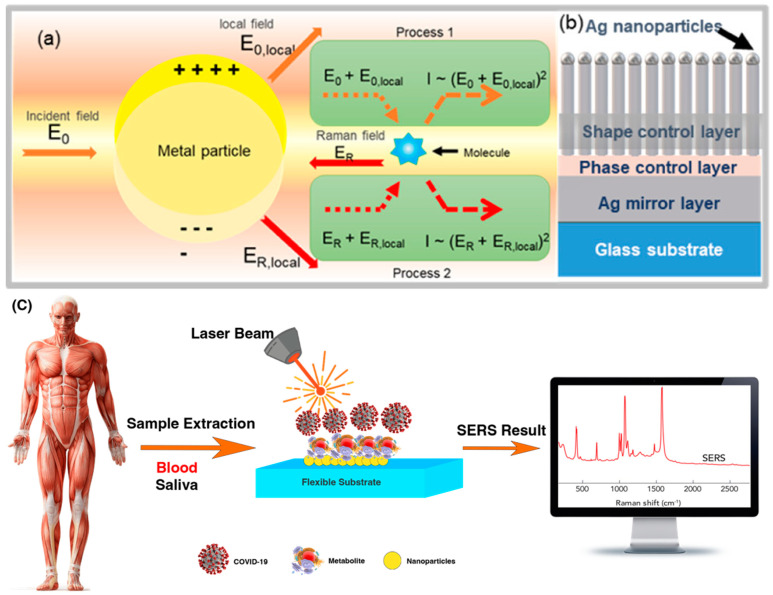
Schematic shows (**a**) mechanism of the surface−enhanced Raman scattering (SERS) electromagnetic (EM) effect, electromagnetic SERS enhancement, and (**b**) multilayer thin−film “local plasmon resonators”. Reprinted with permission [64]. Copyright © 2020 American Chemical Society. (**c**) Principles of COVID−19 detection from biopsies with SERS.

Theoretically, when a molecule is engraved on the surface of gold, silver, or other precious metals, the spectrum of the adsorbed molecule is reduced, resulting in a relaxation of Raman’s selection rules; as a result, more frequencies are seen than in conventional Raman spectroscopy, as shown in Figure 2. The classical theory of optical scattering provides a qualitative concept for the SERS process. Since the most important feature of metal nanoparticles is their optical property, which is not reflected in the size of their mass., metallic nanoparticles typically absorb and scatter light at different frequencies depending on their size, shape, and material. Surface plasmons are excited in small metal particles, but on smooth metal surfaces it is not possible to excite surface plasmons directly. This phenomenon is the best effect in observing the light adsorption of SERS [52]. Among the auxiliary factors in SERS’ optical absorption during COVID-19 diagnosis, we can mainly mention five factors: size, shape, constituents of nanoparticles, interparticle distance, and refractive index of the nanoparticle environment. From the optical point of view, the results of increasing polarization are very important. One of them is the simultaneous increase in scattering efficiency and light absorption by the metal nanoparticles [53]. The incident light beam induces an oscillating dipole (μ) in the particle. The particle scatters the light with oscillating bipolar frequencies. The bipolar moment is generally composed of different coordinated-frequency components. Due to the intensity of the Raman spectrum induced, which is proportional to the quadratic moment, increasing the polarization of the molecule (molecular effect) and increasing the external field acting on the molecule (field effect) are two ways to increase the Raman spectrum [54]. Theoretical models also provide for two main types of excitation: (1) irradiating matter molecules with light, i.e., applying the electric field of light (external field) to the molecule, a larger alternative field is generated near the metal surface due to electromagnetic resonance, which is called the electromagnetic effect. (2) Another method to enhance the Raman spectrum is the chemical effect (molecular effect), in which the polarizability of the molecule is enhanced by the interaction between the molecular surface and the metal surface [55]. The effect of synthesis methods on the ultra-responsive detector ensures that the molecules desired for the detector can be attached to the surface of the metal substrate or at least placed in its vicinity. Therefore, Raman-signal amplification is provided by pulsed resonances in the metal substrate [56].

In the electromagnetic effect, the irradiation of the metal surface with incident light excites the embedded surface plasmons. By tuning the frequency of the incident light with the plasmon frequency (ωp) of the metal, the electric field is enhanced and maximized. Thus, the increase in intensity of the Raman spectrum for the species adsorbed on the metal surfaces is due to the enhancement of an electric field near the metal surface. For scattering to occur, the plasmon oscillations must be perpendicular to the surface. If the oscillations are parallel to the surface, scattering will not occur. For this reason, uneven surfaces or nanoparticle arrays are often used in SERS experiments, because these surfaces create a region where alternating group oscillations can occur [57]. Incident light on the surface can cause a variety of phenomena on the plane. If the surface is such that its properties, such as dimensions and smoothness, are smaller than the wavelength of light, then only the contribution of dipole radiation is considered, and the dipole set contributes to the plasmon oscillations, which enhances the Raman spectrum. The complexity of the SERS effect is due to the fact that the field amplification occurs twice. First, the field enhancement increases the intensity of the incident light, which excites the Raman modes of the molecule under study. This increases the Raman-scattering spectrum. The Raman spectrum itself is enhanced by a process similar to the process of increasing the intensity of the incident light, further increasing the Raman output spectrum. In both phases, the electric field is enhanced by E^2^, so that the final amplitude of the spectrum is proportional to E^4^ [58,59]. The amplitude of the Raman spectrum is not the same for all frequencies. At frequencies where the Raman spectrum has a small shift with respect to the incident light, both the incident laser light and the Raman spectrum may be close to the plasmonic frequency resonance, enhancing E^4^. However, when the shift is large, the incident light and Raman spectrum cannot fluctuate with ωp, so the gain in both phases cannot be maximal [60]. The material from which the metal surface is selected is determined by the resonant frequency of the plasmon. Visible and near-infrared light is used to evoke Raman modes, and is usually of gold and silver for the desired metal surface used in the SERS test. Since the plasmon-resonance frequencies of gold and silver are in this wavelength range and can amplify the electric field of visible and near-infrared light, the absorption spectrum of copper is also in the range that can be used in SERS experiments [61]. Platinum and palladium nanostructures also have plasmonic resonance in the near-visible and infrared regions [62]. Although the electromagnetic model or the electromagnetic effect can explain several properties associated with SERS, it cannot express some specific properties of SERS. When using the electromagnetic model, the chemical structure of the analytical species is not investigated, so the model or chemical effect is also considered. One of the important processes that increases molecular polarization is the mechanism of charge transfer or the chemical effect between the metal and the material (analyte) adsorbed on the metal. The chemical effect occurs simultaneously with the electromagnetic effect, but is used only in special cases [63].

### 2.4. SERS Measurements

Dry or wet measurements of SERS on solid substrates are possible. The dry method is the most common and is perform after the sample has evaporated from the substrate. By immersing the substrate in the sample for a certain period of time (the incubation process), the evaporation process is initiate. Methods of wet experiments include placing a drop of the liquid sample on the substrate SERS or using complex microfluidic systems lab-on-a-chip surface-enhanced Raman spectroscopy (LoC-SERS) on a glass substrate and covering it with a thin coverslip. They can also be perform using the “droplet approach”, where the signal is detected at the edge of a sample droplet on the SERS substrate. Therefore, these approaches affect the final results of SERS due to their drawbacks. When the sample evaporates, the absorption process is abruptly stope, and there is heterogeneity in the generated layer or the “coffee ring” effect. In addition, the metallic nanoparticles may oxidize or dissolve during incubation, resulting in the loss of the perpendicular arrangement of molecules in Raman observations. Inefficient Raman signals arise from the use of different interfaces or the effect of different refractive indices, when a coating glass is used in a liquid environment [65]. The “droplet approach” has the advantage that a suitable change in the hydrophobicity of the substrate can lead to a pre-concentration of the analyte, bypassing diffusion limitations and enhancing the SERS signal. However, for recording measurements by a camera integrated into a Raman spectrometer, it is important to create an ideal distance from the droplet boundary to its center. This distance must be sufficient, to prevent the sample from evaporating at the intended location throughout the experiment, but not so short that the substrates do not respond well [66].

## 3. Flexible SERS Substrate

The best flexible SERS substrates are simply those that support the strongest plasmon resonances; in other words, those that provide the strongest amplification or gain. Metallic nanostructures can amplify the optical field (hotspots) and act like an antenna. When molecules or nanoparticles are in the hotspot, Raman scattering increases dramatically [67]. Most flexible SERS substrates are designed for excitation in the visible infrared range (about 400–1000 nm), the usual range for molecular Raman-scattering experiments. Structures with dimensions below the wavelength range and typically less than 100 nm are the most suitable flexible substrates for SERS. Generally, a good upgrade rate is obtained from structures made of gold or silver. These two metals, gold and silver, are the most commonly used metals in SERS and plasmons [68]. This is due to their good optical properties for the realization of plasmonic resonances in the visible infrared range (400–1000 nm), which is the range of interest for SERS. In principle, there are no limits to the small size of the metal components that make up the flexible SERS substrate. For example, a simple, rough metal surface can also be used as a flexible SERS substrate. However, much less enhancement is achieved than with conventional metal nanostructures. SERS can be measured on structures made of a variety of metals, such as copper or platinum, but even in this case, less enhancement is achieved than with the commonly used metals, gold and silver. Enhancement is not the only important property of a flexible SERS substrate. Among the various aspects, the surface area and surface roughness are of particular importance. SERS is a surface-spectroscopy method, and it is obvious that the surface properties of the flexible substrate play an important role. Surface roughness is an important and crucial factor in SERS experiments and increases the surface sensitivity, resulting in an inelastic scattering of light [69], because at low concentrations it is limited by the inherent strength of the signals from the molecules SERS. However, in situations where the molecules are bound to the flexible substrate by direct contact with the metal, the maximum Raman signal available is limited by the maximum number of molecules in that layer. If the molecule in question is a weak Raman scatterer and the maximum achievable SERS signal is too low, the SERS signal cannot be measured. There are several ways to avoid this problem: using a flexible substrate with higher average gain (which increases the average SERS signal of each molecule), using a flexible substrate with larger surface area (which increases the number of signaling molecules), and increasing the laser power. In addition to these basic features, the simplicity and cost of sample fabrication and preparation should also be considered. The interactions between the flexible substrate and probe play an important role in SERS, so it is useful to use special flexible substrates (or even substrates designed specifically for this molecule) for a certain type of molecules [70]. Flexible SERS substrates can be experimentally divided into three main categories: (1) metal particles (usually nanoparticles) in solution, such as colloidal solutions [71]; (2) flat metal structures; e.g., arrays of metal nanoparticles lying on a flat surface (e.g., glass, silicon, or metal) [72]; (3) metal electrodes [73]. With these interpretations, it can be said that a flexible SERS substrate is a kind of naming for any structure that provides plasmon resonance, which can lead to the development of suitable amplifications in the Raman range and can include metal particles in colloidal solutions; two-dimensional metal structures, such as an array of metal nanoparticles lying on a two-dimensional substrate of glass, silicon, or metal; or metal electrodes [74]. Table 2 shows a comparison of the SERS enhancement of a normal substrate with a flexible substrate.

## 4. Detection of Analytes

**Figure 3:**
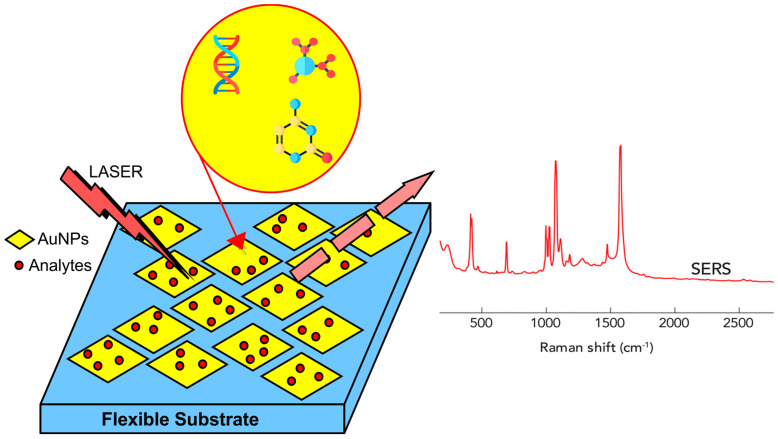
Surface-enhanced Raman spectroscopy (SERS) is a specialized kind of Raman spectroscopy, in which the analyte of interest interacts with gold or silver nanostructures to greatly boost the Raman signal. It allows Raman spectroscopy, a method typically used for identifying analytes, to be used for the detection of trace levels of potentially hazardous or physiologically significant substances.

SERS can be used for direct or indirect detection of analytes. Adsorption of elements, such as antibodies, aptamers, or similar molecules stabilized on nanostructured surfaces, the use of molecular linkers held close enough to the substrate, or adsorption of analytes on the substrate, are direct measurement requirements. This approach is suitable for analytes with a large cross section for Raman scattering, as shown in Figure 3. It benefits from the thorough control and precision of the quantification procedure, as well as the ability to identify and chemically characterize the analyte by examining its binding properties [81,82]. By correlating changes in the spectrum of the SERS metabolites, reaction products, or reporter molecules (RM), indirect-detection measurements of the concentration of the target analyte are made. This approach allows for the detection of analytes with few or no Raman-vibrational modes as well as for multiplex detection. The most common approach for indirect detection in biological samples is the use of reporter molecules. In this approach, substrates are functionalized with one or more molecules (monoplex or multiplex detection) that undergo a change in Raman cross section, upon contact with the target analyte. RMs are usually tiny molecules with large Raman cross sections. Not only are they photochemically stable, but only a small number of their bands overlap with the matrix or analyte, due to limitations in the Raman spectrum [83]. 

### 4.1. Determination of Viruses by SERS

**Figure 4:**
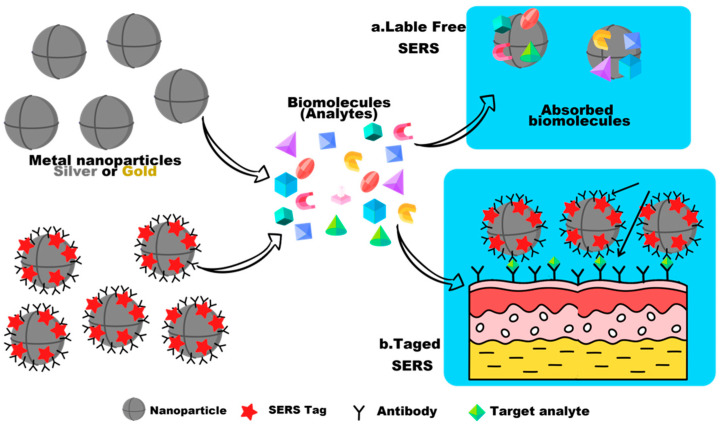
The figure illustrates the differences between a label-free SERS technique and a label-based SERS approach. In label-free SERS, the spectroscopic signal arises from all analytes that adsorb on the SERS substrate (direct detection), while in SERS, the spectroscopic signal results from the labels on a SERS tag that selectively attach to a target analyte (indirect detection).

The combination of reporter molecules (indirect detection) with a sandwich immunoassay is a more sensitive and specific method for virus detection by SERS. In this way, the decision technique is usually composed of the following elements: (1) a SERS tag consisting of a Raman-reporter molecule and an identification element consisting of a specific antibody (detection antibody) entrapped on the active SERS nanoparticles, and (2) a template called the adsorption substrate (which does not have to be a metal surface) functionalized with a binding antibody (an adsorption antibody) to bind the antigen SERS-tag complex. Using the SERS tag, quantification is performed by monitoring the Raman signal from the reporter molecule, before and after the interaction with the absorbing element. The limited specificity and inherent limitations of the direct detection of biological analytes, such as the low affinity for common noble-metal SERS substrates, small scattering cross sections, and large molecule sizes, can be overcome with this technique, as shown in Figure 4 [84].

### 4.2. Multiplex Analysis for Coronavirus Detection

**Figure 5:**
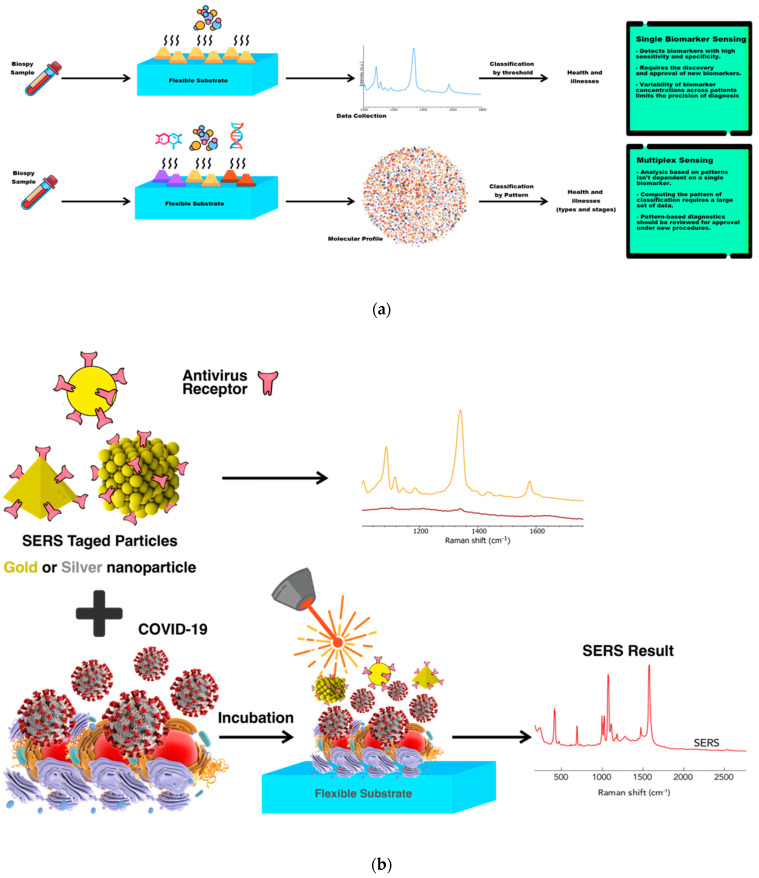
(**a**) Advancing from single−biomarker sensing to multiplex sensing. Diagnostic screening of patient−derived biopsies with multiplex sensors demonstrates high sensitivity and specificity in comparison of single sensors. (**b**) Principles of multiplexed detection using the surface−enhanced Raman scattering (SERS) technique, with metal nanoparticles that are different in size and shape and have unique Raman signals with narrow peaks, thus, they could be used as SERS tags. In addition, schematic illustration of simultaneous detection of COVID−19-associated antigen expressed by SERS imaging.

Multiplex analysis is considered the gold standard for COVID-19 identification, because of its ability to replicate small amounts of the coronavirus’ genetic material. Currently, multiplex testing for SARS-CoV-2 is usually performed on the specimens collected with a swab from the upper-respiratory tract. In addition, several studies have been performed using serum, feces, or ocular secretions [85]. Recently, the Rutgers Clinical Genomics Laboratory developed the (TaqPath COVID-19 Combo Kit) RT-PCR using saliva samples collected from the patient. This method is faster and less painful than other methods of sample collection, reduces potential risks to treatment personnel, and increases sample size [86]. As shown in Figure 5, multiplex analysis begins with the conversion of genomic viral RNA to DNA by DNA-dependent DNA polymerase (RNA-reverse transcriptase). This reaction is designed based on small DNA-primer sequences that specifically identify complementary sequences in the viral RNA genome for reverse transcriptase, to synthesize a short complementary DNA copy (cDNA) of the viral RNA. This is done using a fluorescent dye or a probe labeled with a fluorescent molecule and a quenching molecule, as in the TaqMan method. This is an automated system then repeats the amplification process for approximately 47 cycles, until the viral cDNA is normally detected by a fluorescent or electrical signal [87]. Multiplex analysis is usually performed in a one- or two-step procedure. In the one-step multiplex analysis of the entire reaction, from cDNA synthesis to propagation, the multiplex is performed in a single microtube. In the two-step method, cDNA synthesis and proliferation are performed in separate tubes. Although the two-step method is more flexible and sensitive than the one-step method and also requires fewer raw materials to initiate the reaction, the one-step method is the preferred method for detecting COVID-19. Since it can be started quickly and requires little sample management, the risks of pipetting and contamination during the reverse transcription and proliferation phases are lower [88]. To date, most molecular diagnostic experiments have used multiplex technology targeting different regions of the coronavirus genome, including the ORF1b or ORF8 regions and the nucleocapsid (N) genes, spike protein (S), DNA-dependent RNA polymerase (RdRp), and envelope protein (E). Assays targeting the E envelope protein have been shown to be similar to other coronavirus strains. On the other hand, the low similarity of the N, RdRp, and S genes of coronavirus to those of other bat viruses has made these genes specific targets for identification. Several methods have been used in laboratories to increase diagnostic sensitivity, by examining multiple genes simultaneously or identifying different regions in the same target gene. In general, multiplex methods with high sensitivity and specificity, as well as the ability to process a large number of samples, are the most common methods for detecting COVID-19. Therefore, considering that COVID-19 is likely to remain in the population like influenza viruses, a multiplex-testing method for multiple diseases should be considered as a routine test in the future [89].

### 4.3. SERS COVID-19 Detection

**Figure 6:**
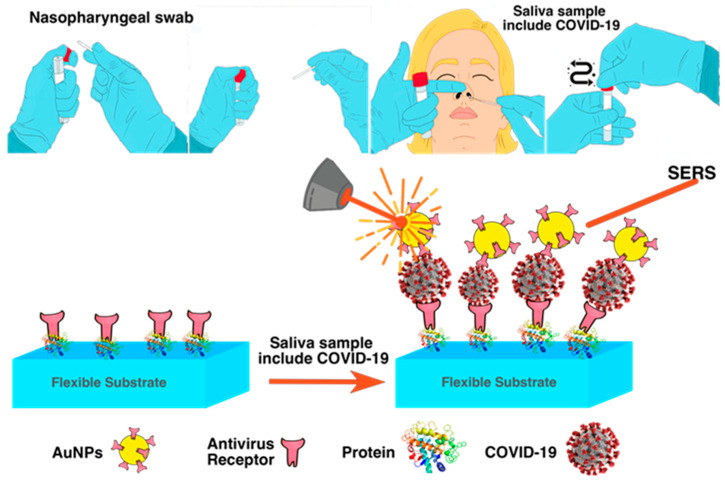
A big swab put into the nasopharynx is passed around in a circle at least four times for a total of 15 s to collect samples of nasal secretions from both nostrils, and mechanism of SERS for COVID-19 detection with saliva sample, which contains COVID-19 virus.

Currently, the analytical approach for diagnosis of the SARS-CoV-2 virus is specific detection by RT-PCR on respiratory specimens, such as nasopharyngeal swabs, oropharyngeal swabs, tracheal aspirates, or alveolar bronchial lavage, as shown in Figure 6. Samples of nasal secretions from both nostrils are collected with a large swab inserted into the nasopharynx by passing it around in a circle at least four times for a total of 15 s. In this approach, two different amplification sections of primers or RNA-dependent RNA polymerase (RpRd) genes, nucleoprotein (NP protein), and envelope protein (E protein) are used. However, the nucleic-acid test has the disadvantage of requiring a high risk of viral RNA degradation during collection, transport, and storage, as well as large amounts of good quality viral RNA (which varies greatly from person to person). Moreover, despite the high sensitivity of the PCR technique in patients with high clinical suspicion, a negative nucleic-acid test does not rule out SARS-CoV-2 infection [90]. When a negative nucleic-acid test result is detected once or twice, other diagnostic options such as serological ELISA tests for IgG and IgM antibodies, formed with bat coronavirus nucleoprotein, must be explored. One of the new alternative techniques used to quantify SARS-CoV-2 is SERS. Zhang et al. investigated the diagnosis of SARS-CoV-2 using SERS in combination with multivariate-statistical analysis. A functional receptor for the human coronavirus spike glycoprotein SARS-CoV-2 in its S1 component is named ACE-2. ACE-2, thus, performed a dual role as a reporter molecule and molecular-recognition element, as its Raman signal was suppressed at a stimulation wavelength of 780 nm after the recognition and binding of the receptor-binding domain (RBD) of the SARS-CoV-2 spike protein. The ACE-2@SN-SERS assay was used to analyze multiple real-water samples from hospitals and pipelines before and during various biological-wastewater-treatment methods, in situ and without pretreatment. A real-time polymerase-chain reaction (PCR) was previously used to detect the presence or absence of SARS-CoV-2 viruses in the samples [91].

Based on the attenuation of the induced SERS signal in the presence of the virus, the detection of the SARS-CoV-2 spike protein in water samples is performed using two markers to classify positive and negative samples. The first indicator is based on the behavior of the band at 1182 cm^−1^, which is associated with the amide II band, for the N-H bending and C-N stretching of the ACE-2 enzyme. In the presence of the SARS-CoV-2 protein, the band changes to 1189 cm^−1^, which is due to a change in the structure of ACE-2 upon binding. Therefore, the ratio of Raman intensity at 1182 cm^−1^ to 1189 cm^−1^ (ratio 1189/1182) was used as a biomarker. The second indicator, which was determined by doing a principal-component analysis with linear-discriminant analysis (PCA-LDA) on the total spectral changes of the data, utilized the first LDA score (LD1 score) to distinguish the positive and negative groups. The results of the two indicators indicated that the detection of the SARS-CoV-2 spike protein was equivalent to that of real-time PCR, with the exception of the samples handled biologically where PCR failed to identify the virus, in contrast to the SERS results of the 1182/1189 ratios and the LD1 score. This was explained by the SARS-CoV-2 spike protein having greater resistance to disinfection compared to RNA. This approach resulted in either a positive or a negative finding; therefore, no LOD value was reported. On the other hand, the lack of a study investigating the selectivity against other ACE-2 target viruses or interference, as well as the fact that the assay reacts to viral coatings or free-spike proteins, which may lead to overestimation of the presence of SARS-CoV-2, are some limitations of this method [92,93]. SERS based on COVID-19 detection and its limitations are shown in Table 3.

## 5. Future and Perspectives

Specifically, for the detection of COVID-19 virus, the preparation of a flexible SERS substrate is emphasized, and its substrate design is a critical factor because no SERS substrate is capable of detecting viruses. Therefore, there is no need to perform SERS testing in the BSL-3 laboratory using the appropriate SARS-CoV-2 pseudovirus. Reconciliation of positive and negative identification with subsequent IVD applications and EUA regulations is possible through the SERS system. To assess the high demand during the disease outbreak, flexible SERS substrates for virus detection should be manufactured on a large scale in the near future, due to the advancement of modular-design technology and high-performance production.

## 6. Conclusions

COVID-19 is a pandemic disease, and because progression of coronavirus infection can lead to severe respiratory problems and possibly death, there is an urgent need for various diagnostic strategies for early detection of the disease. The technique of culturing microorganisms and using a microscope are essential for a deep understanding of the new coronavirus and its relationship to host cells, which is considered one way to detect the virus. However, in the case of the COVID-19 pandemic, this method is very time-consuming and costly. Therefore, to identify different subtypes of the new SARS-CoV-2 coronavirus, the flexible SERS-substrates strategies used in this study were analyzed. According to the new SARS-CoV-2 virus, its quantification was performed using flexible SERS substrates along with multivariate-statistical analysis, by detecting the binding domain of the spike-protein receptor. In this regard, it can be concluded that indirect SERS procedures, especially those based on SIA, have good potential for gaining a foothold as a point-of-care technology for the detection of viral infectious diseases such as coronavirus. Likewise, new analytical strategies for the future are being understood, such as multiplex assays that combine the diagnosis of RVsZO with specific biomarkers or biomarkers of secondary disease due to the progression of infection. The above configuration can be used as a test to screen a patient’s health status or as a dual-confirmatory test.

## Figures and Tables

**Table 1:** Examples of SERS substrates based on their characteristics.

SERS Substrate	Enhancement Factor (EF)	Analyte	Limit of Detection (LOD)	Ref.
Ag nanoparticles/microporous silicon	-	rhodamine 6G	10^−9^ M	[29]
Au nanothorns/macroporous silicon	10^8^	crystal violet	10^−12^–10^−15^ M	[30]
Ag nanoparticles/silicon nanopillars	10^11^	acetone	0.0037 ng	[31]
Au nanoparticles/mesoporous silicon	-	benzenethio	10^−6^ M	[32]
Ag nanoparticles/mesoporous silicon	2.8 × 10^8^	p-thiocresol	5.2 × 10^−9^ M	[33]

**Table 2:** Comparing the SERS enhancement of a normal substrate with a flexible substrate.

Types of Flexible SERS Platforms	Compositions	Unique Features including Flexibility	Detection Limit	Fabrication Methods	Applications	Ref.
In situ detection for SERS	Ag NPs	Floating metal film	10^−11^ m (4-ATP)	One-step electronic reduction	Liquid-phase detection	[75]
	Ag/Au nanowires	3D cross-point nanostructures	10^−11^ m (R6G)	Nanotransfer printing	Glucose detection	[76]
Actively tunable SERS	Au NPs	An open-to-closed system	-	Cast method	Bio-macromolecules’ detection	[77]
	Wrinkled graphene/Au NPs	50% tensile strain without performance degradation	10^−9^ m (R6G)	Graphene transfer/physical deposition	Multiple analytes’ detection	[78]
Swab-sampling approach	Au NPs	“Sticky” feature	0.24 ng cm^−2^ (Thiram)	Drop-dry method	Pesticide residues’ detection	[79]
	Ag NWs	High capture capability of pesticides	40.2 ng cm^−2^ (PQ)	Mixing and vacuum filtration	Onsite residual-pesticide detection	[80]

**Table 3:** SERS based on COVID-19 detection and its limitations.

Target of Virus	Technique	Material Coating	Diagnosis of COVID-19 in Clinical on Surfaces		Limit of Detection	Limitations	Ref.
COVID-19	SERS microfluid	Au/Ag	✓	✗	NA	● Weak signal relative to background. ● Low sensitivity with low protein concentration. ● Consumes time to collect sample. ● Laser wavelength is unstable.	[12]
MERS	SERS-LSPR	Silver nanodot	✓	✗	1–10^6^ nM		[94]
COVID-19	SERS	Gold nanoparticles	✓	✗	17.7 pM		[95]
COVID-19	SERS-LSPR	Silver nanodot	✓	✗	153.53, 230.37 pM		[96]
COVID-19/spike protein	LSPR	AuNIs	✓	✗	0.22 ± 0.08 pM	● Mass transport challenge. ● Heterogeneity of surface.	[97]
COVID-19 RNA	Fluorescence	Gold	✓	✗	1000 TU mL^−1^	● Collection of sample consumes time. ● Low sample size.	[98]
Coronavirus/N-protein	SPR	NA	✓	✗	2.17 nM	● Low selectivity. ● A small perception depth. ● Mass transport challenge. ● Heterogeneity of surface. ● Misinterpretation of data.	[99]

✓: Yes; ✗: No.

## Data Availability

All data generated or analyzed during this study are included in this published article.
